# How dietary intake of type 2 diabetes mellitus outpatients affects their fasting blood glucose levels?

**DOI:** 10.3934/publichealth.2019.4.424

**Published:** 2019-10-21

**Authors:** Nguyen Thanh Ha, Nguyen Thi Phuong, Le Thi Thu Ha

**Affiliations:** Ha Noi University of Public Health. No. 1A Duc Thang Ward, North Tu Liem, Ha Noi, Vietnam

**Keywords:** diabetes type 2, blood glucose, food consumption, food habit, meal frequency, Vietnam

## Abstract

**Objective:**

The study aims to explore the association between the adherence to dietary recommendations among type 2 diabetes mellitus (T2DM) patients with their fasting blood glucose levels.

**Method:**

This is a cross-sectional anthropometric and dietary study conducted on 169 T2DM patients receiving outpatient treatment at the Central Nursing and Rehabilitation Hospital in Thanh Hoa provincial hospital in Vietnam in 2018.

**Results:**

The rate of patients who had good fasting glycemic control was still low (30.8%). Their diets were poor in energy and unbalanced; the contribution of carbohydrates to the total dietary intake was high (68.2%), and only 24.9% of patients consumed 4–6 meals/day. There was a statistically significant association between the dietary intake with carbohydrate dietary intake containing ≥60% carbohydrate and the number of meals per day (<4 meals) with their fasting blood glucose levels (OR = 4.964, p < 0.05 and OR = 16.508; p < 0.01, respectively).

**Conclusions and recommendations:**

Hospital staff are advised to combine treatment with dietary counseling to help patients controlling their weights and glycemic levels more efficient, thereby contributing to improving their treatment outcomes.

## 1. Introduction

Type 2 diabetes mellitus (T2DM) is one of the leading public health issues worldwide. According to a WHO's report in 2018, 135 million peoples suffered from this disease in 2010, and in the year of 2017, it increased 1.5 times compared to 2010. The prevalence of diabetes mellitus is expected to rise from 425 million to 629 million people by 2045 [1]. In Vietnam, 20 years ago, diabetes sufferers accounted for 1–2% of the national population, most of whom lived in major cities. However, by 2012, the national figure was estimated to be 5.7% [2]. Diabetes mellitus causes serious consequences to individuals, families and the entire society, such as heavy healthcare cost burden, reduced productivity, increased mortality rates, and low life expectancy [1],[2]. Proper diets, i.e. adequate and good-quality diets, support moderate glycemic control, maintain proper weights help reducing complications and improve patients' quality of life [3],[4]. Certain studies worldwide indicate statistically significant associations of proper dietary practices with the fasting glycemic level and the HbA1c level in diabetic patients [5]–[7]. Some other studies pointed out that the overconsumption of carbohydrates, cereals and fast foods had a positive statistical significant association with fasting glycemic levels [8]–[12]. Interventional studies also proved that energy-reduced and low-carbohydrate diets help control the blood lipid and glucose levels [10],[13],[14].

In Vietnam, many studies have been conducted on diabetic patients, mainly focusing on patients' adherence to treatment, or simply describing their nutritional status, dietary intake and eating habits [15]–[19]. Their results shown that the patients' dietary intakes were improper, most of which contained lower amounts of energy than the Recommended Dietary Allowance (RDA). A high proportion of patients had deficient lipid but excessive carbohydrate intakes. Those studies also showed a fair proportion of patients suffered from overweight/obesity and had various eating habits. Particularly, some patients were active in avoiding carbonated drinks, cakes, sweets and other confectionary products, as well as animals' internal organs; nevertheless, they did not know which foods they should choose, for example those rich in fibers and plant-originated fats [15]–[19]. However, the research on associations of diabetic patients' dietary intakes and eating habits with their glycemic levels have not been given much attention in Vietnam.

This article aims to describe the association between the dietary intake of T2DM outpatients in the Central Nursing and Rehabilitation Hospital in Thanh Hoa Province and their fasting glycemic levels, thereby providing additional scientific evidence to recommend patients to practice proper diets in order to contribute to improving treatment outcomes.

## 2. Materials and method

### 2.1. Study design

This descriptive cross-sectional study took place between December 2017 and July 2018 on 18 to 75-year-old T2DM patients who received outpatient treatment for at least three months up to February 2018 and did not use insulin. It was conducted at the Central Nursing and Rehabilitation Hospital in Thanh Hoa province (where rehabilitation, nursing and healthcare services are provided for those who have demands). Patients were excluded from the study if they had comorbidities, such as bacterial infections, contagious diseases, cancer, diabetes-related complications leading to amputation or renal complications or lacked of consciousness.

### 2.2. Sampling

On average, the hospital received 60 T2DM patients coming over for routine health checks every month. The research team recruited all patients seeking healthcare services at the hospital within three months (from February 2018 to April 2018) to the study. One hundred and sixty - nine patients were eligible and voluntarily participated in the study.

### 2.3. Data collection

Data were collected from February 2018 to April 2018 when patients had routine health checks at the hospital. Investigators collaborated with nurses at the outpatient department in asking the patients to rest for about 15 minutes before measuring their blood pressure with mercury blood pressure monitors. Patients had their blood taken for fasting blood glucose tests and had their weights, heights, waist and hip sizes measured. Then, they were interviewed about their eating habits and frequencies of consuming certain types of foods that are recommended or not recommended for diabetic patients within one month prior to the time of the survey, and 24-hour diet recall interviews. During these interviews, investigators noted down the names of all foods that patients consumed one day before the interviews, and other information, such as the number of meals, the amount of foods consumed for each meal (including drinks), and food processing at home and outside the home (for example at restaurants). Investigators showed photo albums to patients to help them recall and estimate the amount of foods they consumed as exactly as possible. On the day before the interview, if any patient ate when attending parties (like wedding or death anniversary parties), he or she would be excluded from the study.

By the end of each day, all questionnaires were cross-checked in order to identify and timely recollect missing information.

### 2.4. Measures

#### 2.4.1. Outcome variables

The fasting blood glucose is considered to be high if the fasting serum glucose level exceeds 7.2 mmol/L [20].

#### 2.4.2. Explanatory variables

- Demographic information of the study participants covered age, gender, occupation, educational level and the duration of diabetes treatment.
- Body mass index (BMI): according to the WHO classification of BMI, a healthy BMI ranges between 18.5 kg/m^2^ and 24.99 kg/m^2^, and overweight/obesity is defined with a BMI ≥25 kg/m^2^ [21].
- A normal waist circumference (WC) for females is <80 cm, while a high WC is ≥80 cm. In males, a healthy WC is <90 cm, and a high WC is ≥90 cm [22].
- An ideal waist-to-hip ratio (WHR) is <0.9, and a high WHR is ≥0.9 for males. For females, the ideal WHR is <0.8, whereas a WHR ≥0.8 is considered to be high [22].
- Blood pressure: blood pressure (or hypertension) is considered to be high (or raised) when a systolic BP is equal to or above 140 mm Hg, and/or a diastolic BP is equal to or above 90 mm Hg [23].
- Dietary energy: According to the dietary guideline of Vietnam's Ministry of Health, the total dietary energy intake recommended for T2DM patients at rest and while engaged in light physical activity is set at 30 kcal/kg ideal body weight (IBW)/day. Ideal body weight (IBW) is calculated by multiplying BMI by [height]^2^; (in this study, BMI = 22 kg/m^2^ for males and 21 kg/m^2^ for females) [24],[25].
- A dietary intake is regarded as balanced when carbohydrates, lipids and proteins contribute to 55–60%, 20–25% and 15–20% of the total dietary energy, respectively [24],[25].
- The frequency of consuming foods recommended and not recommended for diabetic patients within one month prior to the study was divided into four levels (never, rarely, occasionally and constantly).
- Patients ate 4–6 small meals per day.

### 2.5. Data analysis

The 24-hour dietary recall data were cleaned and converted from cooked food to clean raw food according to the conversion table of the Vietnam National Institute of Nutrition. The nutritional value was calculated based on the Vietnamese food composition table 2017 and compared to Recommended Dietary Allowance (RDA) for Vietnamese people in 2016. Food frequency data was cleaned and entered into excel to calculate the frequencies of regular, occasional, rarely and never consumption. Data on demographic characteristics and eating habits randomly checked to ensure their accuracy.

Frequencies and proportions were used to describe diets and nutritional status of the study participants. Univariate and multivariate logistic regression analyses with “Enter” method (enters all variables into the equation at the beginning) was used to explore the associations of independent factors with the patients' fasting blood glucose levels.

### 2.6. Ethical considerations

The study was approved by the Ethical Committee of Hanoi University of Public Health *(according to Decision No. 059/2018/YTCC-HD3 dated February 27, 2018 of IRB–Hanoi University of Public Health)*. The research team obtained approval to conduct the study from the Hospital Director Board before undertaking data collection. Each participant was given a written consent form and asked to read it carefully. An interview only started only if the participant sign the written consent form. Participants could withdraw from the study at any time without any consequences. At the end of each interview, the investigator provided the participant with a counselling session about nutrition for diabetes patients. The study results would then be disseminated to relevant stakeholders to inform policies and interventions for improving the health of diabetic patients and pave the way for future studies.

## 3. Results

Table 1 shows that male patients outnumbered female patients (52.1% vs 47.9%, p > 0.05), and the age of the study participants was 61.6 years. Most participants completed primary education (37.9%), and those who were illiterate and did not complete primary education accounted for a high proportion of 5.9%. About one third of all participants were self-employed people (32.5%) and 26.6% of them were housepersons. Patients receiving diabetes treatment for >5 years made up 20.7% of all participants. With regard to nutritional status, their mean BMI was 23.4 kg/m^2^, and the OW/OB rate was 28.4%; Patients with a high WC and a high WHR constituted 27.2% and 47.3%, correspondingly. The hypertension rate was rather high, standing at 60.9%. The mean blood glucose level of the study participants was 8.9 ± 2.8 mmol/L and less than one third of them had normal fasting blood glucose levels (30.8%).

**Table 1.** General information about study participants (n = 169).

	Information	Frequency (n)	Proportion(%)
Gender	Male	88	52.1
	Female	81	47.9
Age	Mean age (X ± SD)	61.6 ± 8.5	
Highest educational level	Illiterate	10	5.9
	Primary school	64	37.9
	Secondary school	52	30.8
	High school	33	19.5
	Secondary technical school/college	6	3.6
	University/Post-graduate	4	2.4
Occupation	Public sector employee	7	4.1
	Private sector employee	1	0.6
	Self-employed (freelancer)	55	32.5
	Houseperson	45	26.6
	Retiree/elderly person	61	36.1
Duration of treatment	<5 years	134	79.3
	≥5 years	35	20.7
Body mass index	Mean BMI	23.4 ± 3.2	
	Overweight and obesity (BMI ≥25)	48	28.4
Waist circumference	High waist circumference	46	27.2
WHR	High WHR	80	47.3
Blood pressure	Hypertension	103	60.9
Fasting blood sugar	Mean blood glucose (mmol/l)	8.9 ± 2.8	
	Mean fasting blood glucose level (<7.2 mmol/l)	52	30.8

Table 2 shows that the 24-hour dietary energy intake of the participants was below RDA in both males and females (1361.5 ± 140.2 kcal/day vs 1319.9 ± 164.3 kcal/day, respectively (p < 0.05). In terms of dietary balance, the rate of the total energy intake from lipids (%) was found below RDA (20–25%) (12.9% in males and 13.0% in females). However, the rates of energy intake from carbohydrates in both males and females (68% and 68.2%, respectively) were above the RDA levels of 55–60%. The study participants consuming 4–6 meals/day formed 24.9%; however, most of them still just ate three meals per day (55.6%).

Figure 1 shows the participants' adherence to consuming recommended foods and avoiding non-recommended ones. A high proportion of patients who usually and sometimes consumed recomended food, except some foods that should be eaten with low usualy consumption rates such as wholegrain rice, black bread, maize, sweet potatoes and cassava. The rates of patients constantly and occasionally consume certain foods recommended for patients with diabetes were high (fish – 94.1%, shrimp – 81.1%, and vegetables – 75.7%).

**Table 2.** Dietary intake of T2DM patients.

Dietary intake		Reality	Recommendations
Mean dietary energy intake	Male	1361.5 ± 150.2	1745.8 ± 154.3
	Female	1319.9 ± 164.3	1551.4 ± 125
	Total	1341.6 ± 158	1652.6 ± 171.0
Energy from proteins (%)	Male	17.7 ± 2.26	15–20%
	Female	17.9 ± 2.1	15–20%
	Total	17.8 ± 2.19	15–20%
Energy from lipids (%)	Male	12.9 ± 4.0	20–25%
	Female	13.0 ± 4.1	20–25%
	Total	13.0 ± 4.1	20–25%
Energy from carbohydrates (%)	Male	68.3 ± 4.9	55–60%
	Female	68.0 ± 5.2	55–60%
	Total	68.2 ± 5.0	55–60%
The proportion of animal fat to total fat intake	Male	54.0 ± 20.1	< 60%
	Female	54.2 ± 18.6	< 60%
	Total	54.1 ± 19.3	< 60%
The proportion of animal protein to total protein intake	Male	46.6 ± 9.8	30–50%
	Female	47.6 ± 11.7	30–50%
	Total	47.1 ± 10.7	30–50%
Number of meals per day (n, %)	<3 meals	33 (19.5)	4–6 meals/day
	3 main meals	94 (55.6)	
	4–6 small meals	42 (24.9)	

Patients were good at adhering to limited consumption of certain foods. More than 90% of patients rarely or never ate any non-recommended foods, such as baked sweet potatoes, sugar/honey, lactose-fermented cabbages/eggplants, salted fish/meat, fast foods, canned foods, internal organs of animals, carbonated drinks and dried fruits.

Table 3 shows that in the univariate models, age, the rate of energy derived from carbohydrates in diet, BMI, blood pressure and the number of meals per day were statistically significantly associated with the fasting blood glucose level in diabetic patients (p < 0.05). However, when those factors were included in the multivariate model, only two factors, namely the dietary carbohydrate intake and the rate of patients with more but smaller meals per day had a statistically significant association with the fasting blood sugar. Patients with >60% of energy intake from carbohydrates faced the risk of having a blood sugar level 4.9 times higher than those who had <60% of their total calories from carbohydrates (p < 0.05). Similarly, the risk of having a high fasting blood sugar level among those having <4 meals/day was 16.5 times higher than those with at least 4–6 meals/day (p < 0.01).

Figure 1. Frequency of consuming recommended foods and non-recommended foods.

**Figure 1a.**
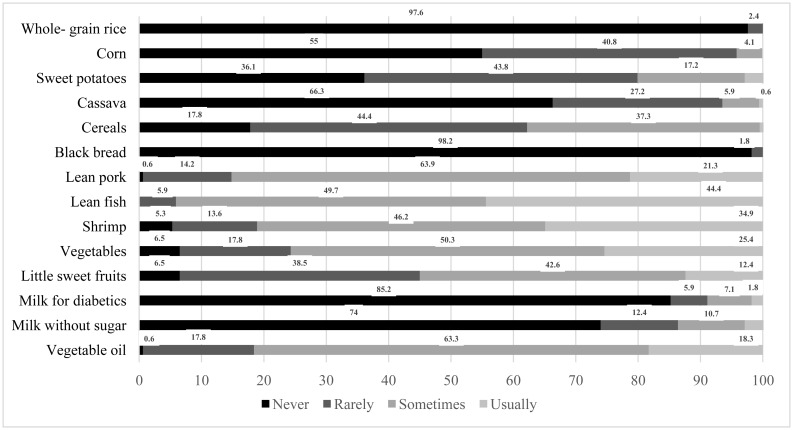
Frequency of consuming recommended foods.

**Figure 1b.**
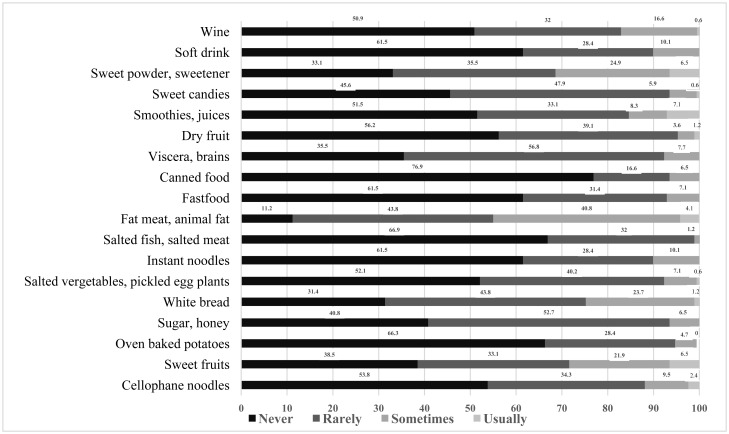
Frequency of consuming non-recommended foods.

**Table 3.** Associations between certain factors with the fasting blood glucose level in diabetic patients.

Factors	Univariate			Multivariate		
	OR	95% CI	p	OR	95% CI	p
Age						
≤60	-			-		
>60	0.457	(0.227–0.921)	0.029	0.654	(0.26–1.643)	0.366
Gender						
Male	-			-		
Female	0.794	(0.413–1.5277)	0.489	0.73	(0.238–2.243)	0.583
Highest educational level						
High school and above	-			-		
Below high school	1.289	(0.618–2.687)	0.499	1.13	(0.398–3.213)	0.818
Duration of treatment						
<5 years	-			-		
>5 years	1.365	(0.589–3.163)	0.468	2.308	(0.733–7.274)	0.153
BMI						
<25	-			-		
≥25	0.443	(0.22–0.893)	0.023	0.869	(0.323–2.336)	0.78
Waist circumference						
Normal	-			-		
Large	1.521	(1.0590–2.56)	0.072	0.397	(0.128–1.235)	0.111
Waist-to-hip ratio						
Normal	-			-		
High	0.686	(0.356–1.321)	0.26	1.461	(0.414–5.155)	0.555
Blood pressure						
Normal	-			-		
High	0.461	(0.226–0.94)	0.033	0.438	(0.163–1.179)	0.102
Dietary carbohydrate intake						
<60%	-					
≥60%	10.455	(2.136–51.162)	0.004	4.964	(1.628–39.237)	0.029
Dietary protein intake						
15%–20%	-			-		
<15% hay >20%	0.504	(0.241–1.053)	0.068	0.46	(0.174–1.211)	0.116
Dietary energy intake						
Adequate (95–100%)	-			-		
Inadequate (<95% & >100%)	1.31	(0.366–4.684)	0.678	1.894	(0.316–11.361)	0.485
Rate of patients consuming more but smaller meals per day						
Adequate (4–6 meals/day)	-			-		
Inadequate (<4 meals/day)	17.1	(7.276–40.282)	0.001	16.508	(6.241–43.664)	0.001

## 4. Discussion

### 4.1. Dietary intake and eating habits of diabetic patients

Dietary control involves reducing the amount of energy intake, having suitable proportions of energy-generated nutrients and choosing foods recommended for T2DM patients. Our study results show that patients had a deficient energy intake, but consumed excessive carbohydrates. This result is consistent with those from some previous studies [17],[18],[26]. A possible explanation would be Vietnamese people are recommended to have 60–65% of dietary energy intake from carbohydrates [27]. This figure is higher than that for those living in other countries where rice is less consumed in daily meals [27]. In addition, the actual carbohydrate intake of Vietnamese people, according to the results of the National Nutrition Survey, is quite high (67–70%) [28]; hence, it is difficult for them to change their eating habits once they are diagnosed with diabetes. Patients might think that they only needed to “eat less”, but they did not know what they needed to do in order to reduce their carbohydrate intakes. The proportion of the total protein and the proportion of animal-originated proteins in patients who strictly adhered to dietary guidelines were acceptable. In the USA, proteins contributed to 15–20% of the dietary energy intake recommended for both healthy people and diabetic patients [3]. Some studies on healthy people and those suffering from type 2 diabetes demonstrated that protein does not increase the serum glucose levels, but make people feel full. This limits further food intake and controls the blood sugar levels [3],[7],[6].

Evidence indicates that the consumption of wholegrain cereals, vegetables, fruits, beans, nuts and dairy products, e.g. yogurt, is beneficial for diabetic patients [7]. In our study, patients consuming foods recommended for diabetic patients accounted for a small percentage. In particular, most of them neither ate wholegrain rice a type of wholegrain cereal (97.6%), and no one had ever consumed yogurt. This may be because our patients had poor knowledge of nutrition, lived in poor economic conditions, or were engaged in self-employed jobs (32.5%). However, among recommended foods, fish, shrimps and vegetables were highly consumed. This may be because this study was conducted in a coastal region; therefore, the rates of inhabitants eating fish and shrimp were higher than those in previous studies [15],[18].

Our study results shown that only 24.9% of patients had 4–6 small meals/day; this figure was lower than in other studies [15],[17],[18]. This may result from the lack of knowledge among our patients who were self-employed. Another possible reason was that being quite busy, most of the patients were unable to prepare their meals themselves. However, having small meals is an essential means of providing enough energy to prevent hypoglycemia; therefore, it is necessary to counsel patients about how to prepare minor meals which are simple, easy to cook and suitable for their situations so that they have enough nutrition.

### 4.2. The association between diets/portions and fasting blood sugar levels

According to the National Institute of Nutrition of Vietnam, the carbohydrate intake recommended for diabetic patients should range from 55% to 60% of the total energy intake [29]. This RDA level is higher than those in some other countries [3],[7],[30],[31]. The study results also shown that carbohydrates contributed to 68.2% of the total energy intake, which was above RDA and was associated with the fasting blood sugar level (p < 0.05). In terms of the metabolic mechanism, diets were associated with blood sugar reactions, including the amounts of carbohydrate intake, the types of sugar consumed (i.e. glucose, fructose, sucrose, or lactose), as well as the preparation and processing of foods [3]. Laboratory research and clinical trials also provide evidence on the effects of carbohydrates on blood sugar reactions. Half of 11 randomized controlled trials in the 2001–2010 period on low-carbohydrate diets conducted by the American Diabetes Association show that the carbohydrate intake <40% contributed to improving HbA1c and blood glucose levels in the intervention group, compared to the control group [7]. According to those studies, in T2DM patients, a diet low in carbohydrates helped reduce blood glucose and triglyceride levels. Therefore, reduced consumption of carbohydrates was recommended to be included in the objective of T2DM treatment [3],[7],[32]. In terms of food choice for a proper diet, foods low in carbohydrates, especially wholegrain cereals, fruits, vegetables and low-fat milk, should be consumed as they help control blood glucose levels. Existing evidence also points out the association between the quality of carbohydrates and blood glucose levels. More precisely, wholegrain cereals and fibers are better choices for T2DM patients than refined cereals [3],[7]. In our study, almost all patients never consumed wholegrain cereals, milk or yogurt. This might also affect their fasting blood sugar levels.

Diabetic patients were recommended to eat small and balanced meals throughout the day (up to six meals per day) to prevent increased levels of blood sugar (hyperglycemia) [3],[7],[29]. The univariate and multivariate analyses in our study showed that patients who had <4 meals/day were at risk of raised fasting blood sugar levels (p < 0.01). Some studies also reported similar results that is the frequency of meals had an effect on blood sugar and serum insulin. Micheal et al. conducted a study in which they assigned a group of patients to have six meals per day and another group to have two large meals per day; the total energy intake was the same for both groups. The results showed that having two large meals resulted in a maximum variation of glucose 84% greater than that in the case of six small meals (6.1 ± 0.5 vs 3.3 ± 0.5 mmol, p < 0.005) [33]. Another study conducted in Greece also compared the six-meal model with the three-meal model in which pre-diabetes patients or those diagnosed with T2DM had the same total energy intake. It pointed out that the six-meal model was better at controlling the blood sugar in obese patients with diabetes and improving and/or stabilizing post-meal blood glucose in pre-diabetic people, compared to the three-meal model [34]**.** A possible explanation for this is that if a type 2 diabetes patient consumed excessive amounts of glucose (especially after a big meal), the insulin secreted by the pancreas was insufficient to transport glucose from the bloodstream into cells. This resulted in heightened levels of glucose in the bloodstream. Therefore, diabetic patients should eat more but smaller meals per day (instead of having fewer but bigger meals), as this would help their bodies have better control of blood glucose levels. Our study provides additional evidence that more meals per day, when keeping the total energy intake unchanged, may benefit diabetic patients or pre-diabetic people in terms of glycemic control.

### 4.3. Limitations

This cross-sectional descriptive study used the 24-hour recall method (1 time). As this method depends on the patients' memories and collaboration, it is difficult to estimate the weights of some foods correctly. The participants only excluded those on insulin therapy while data on other anti-diabetic medications, which might affect blood glucose measures, were not collected in this study. Besides, in this study we could only had fasting blood sugar tests performed-blood sugar readings were only valuable helpful at the time of doing the tests, but had not yet measured HbA1c levels as well as glycemic variability to draw a more comprehensive picture of the average percentage of blood sugar in patients over the past three months. Besides, we did not ask patients about their exercise and physical activity regimes although these factors play an important role in improving insulin sensitivity and sustainably reducing blood sugar. Therefore, further studies should take into consideration and handle the limitations that had not been dealt with in our study.

## 5. Conclusions and recommendations

The rate of patients who were good at controlling their fasting blood sugar was still low (30.2%). Their diets were poor in energy and unbalanced; the contribution of carbohydrates to their dietary energy intakes was high (68.2%), and only 24.9% of patients ate 4–6 meals/day. There existed a statistically significant association of dietary intake with carbohydrates ≥60% and the number of meals per day (<4 meals) with the fasting blood sugar levels (OR = 4.964, p < 0.05 vs OR = 16.508, p < 0.01). Hospital staff are advised to combine treatment with dietary counseling to help patients better control their weights and blood glucose levels, thereby contributing to improving their treatment outcomes.
